# The impact of trained radiographers as concurrent readers on performance and reading time of experienced radiologists in the UK Lung Cancer Screening (UKLS) trial

**DOI:** 10.1007/s00330-017-4903-z

**Published:** 2017-06-22

**Authors:** Arjun Nair, Nicholas J. Screaton, John A. Holemans, Diane Jones, Leigh Clements, Bruce Barton, Natalie Gartland, Stephen W. Duffy, David R. Baldwin, John K. Field, David M. Hansell, Anand Devaraj

**Affiliations:** 1 Department of Radiology, Guy’s and St Thomas’ NHS Foundation Trust, Great Maze Pond, London, SE1 9RT UK; 2 Department of Radiology, Papworth Hospital NHS Foundation Trust, Papworth Everard, Cambridge, CB23 3RE UK; 3 Department of Radiology, Liverpool Heart and Chest Hospital, Thomas Drive, Liverpool, Merseyside L14 3PE UK; 4 Department of Radiology, Royal Brompton Hospital, Sydney Street, London, SW3 6NP UK; 5 Centre for Cancer Prevention, Wolfson Institute of Preventive Medicine, Barts and The London School of Medicine and Dentistry, Charterhouse Square, London, EC1M 6BQ UK; 6 Respiratory Medicine Unit, David Evans Research Centre, Nottingham University Hospitals, Nottingham, NG5 1PB UK; 7 Roy Castle Lung Cancer Research Programme, Department of Molecular and Clinical Cancer Medicine, Institute of Translational Medicine, The University of Liverpool, The William Duncan Building, 6 West Derby Street, L7 8TX Liverpool, UK

**Keywords:** Multidetector computed tomography, Diagnostic imaging, Lung neoplasm, Pulmonary nodule, Mass screening

## Abstract

**Objectives:**

To compare radiologists’ performance reading CTs independently with their performance using radiographers as concurrent readers in lung cancer screening.

**Methods:**

369 consecutive baseline CTs performed for the UK Lung Cancer Screening (UKLS) trial were double-read by radiologists reading either independently or concurrently with a radiographer. In concurrent reading, the radiologist reviewed radiographer-identified nodules and then detected any additional nodules. Radiologists recorded their independent and concurrent reading times. For each radiologist, sensitivity, average false-positive detections (FPs) per case and mean reading times for each method were calculated.

**Results:**

694 nodules in 246/369 (66.7%) studies comprised the reference standard. Radiologists’ mean sensitivity and average FPs per case both increased with concurrent reading compared to independent reading (90.8 ± 5.6% vs. 77.5 ± 11.2%, and 0.60 ± 0.53 vs. 0.33 ± 0.20, respectively; p < 0.05 for 3/4 and 2/4 radiologists, respectively). The mean reading times per case decreased from 9.1 ± 2.3 min with independent reading to 7.2 ± 1.0 min with concurrent reading, decreasing significantly for 3/4 radiologists (p < 0.05).

**Conclusions:**

The majority of radiologists demonstrated improved sensitivity, a small increase in FP detections and a statistically significantly reduced reading time using radiographers as concurrent readers.

***Key Points*:**

• *Radiographers as concurrent readers could improve radiologists’ sensitivity in lung nodule detection.*

• *An increase in false-positive detections with radiographer-assisted concurrent reading occurred.*

• *The false-positive detection rate was still lower than reported for computer-aided detection.*

• *Concurrent reading with radiographers was also faster than single reading.*

• *The time saved per case using concurrently reading radiographers was relatively modest.*

**Electronic supplementary material:**

The online version of this article (doi:10.1007/s00330-017-4903-z) contains supplementary material, which is available to authorized users.

## Introduction

Lung cancer screening using low-dose CT within comprehensive, quality-assured programmes has recently been recommended in both the USA [1–3] and Europe [4], but questions regarding cost-effectiveness [5] and implementation remain. Amongst other considerations, the successful large-scale implementation of such programmes will require an expansion in the number of thoracic radiologists as well as optimisation of CT reading workflow for lung nodule detection. With respect to the latter, computer-aided detection (CAD) software as a second reader for this task has been extensively evaluated and shown to improve sensitivity [6–16].

Despite this extensive evaluation, CAD has not been universally adopted in lung nodule detection, and has also not been prospectively evaluated in any of the randomised controlled trials in CT lung cancer screening. The reticence to use CAD may be due to the need for two rounds of reading by a radiologist when using CAD as a second reader – the radiologist has to first independently read the study, present it to the CAD system, and then re-evaluate the study with the specific aim of assessing the CAD marks, in order to arrive at a final set of agreed findings. This has led to interest in using CAD as a concurrent reader. In concurrent reading, the first round of radiologist reading is removed; instead, the study is processed by the CAD and presented to the radiologist, who accepts or rejects the CAD marks, and finally performs an independent search for any missed nodules. Although this method has undergone only limited evaluation, it has been shown to decrease reading times without compromising sensitivity [17–19].

The use of radiographers (sometimes referred to as technologists) as alternative readers for the task of lung nodule detection, either alone [20] or reading in combination with a CAD system [21], has shown promise, with the performance of some radiographers comparing favourably with that of radiologists. These findings suggest that radiographers could aid radiologists in CT lung cancer screening, but this hypothesis remains untested.

The aim of the present investigation was thus to prospectively compare the performance of radiologists reading CTs independently (i.e. unaided) with their performance when using radiographers as concurrent readers, with respect to sensitivity, false-positive detection and reading times, in an actual lung-screening setting.

## Materials and methods

### Study population and case selection

The UK Lung Cancer Screening (UKLS) trial is a randomised controlled trial evaluating low-dose multidetector CT for lung cancer screening using a single screen design, whereby a single round of screening (i.e. prevalence screening) is performed [5, 22]. Recruitment procedures, selection criteria and screening protocols have previously been published [22]. A brief overview of the study design, along with the study’s ethical approval, CT imaging protocol, nodule classification protocol and CT reading methods are provided in the Supplementary Material.

The current investigation was performed prospectively. Between June and October 2012, the baseline CT studies of 369 consecutive participants in the LDCT arm of the UKLS trial were read for this study.

### CT evaluation by radiologists

Each CT study was first read by a single radiologist at one of the two participating sites, namely Radiologist A at Local Site 1 and Radiologist B at Local Site 2, both with more than 10 years of specialist thoracic imaging experience. The studies were then transmitted to a central reading site for a second independent reading by Radiologist C, with 10 years’ experience, or by Radiologist D, with 7 years’ experience.

### Selection of reading radiographers

Four radiographers who had had experience in thoracic CT scan acquisition, and were able to commit at least 4 hours a week over the study period, were selected as readers. Radiographer 1 read CTs at Local Site 1, and Radiographer 2 read CTs at Local Site 2. Two radiographers (Radiographers 3 and 4) read CTs at the central site. These four radiographers were the same radiographers who participated in our previous investigation comparing them directly with radiologists, and had been trained in CT lung nodule detection as described previously [20]. Briefly, the radiographers had been given an introductory tutorial that covered the principles of low-dose CT, thoracic anatomy and the CT reading method to be used, followed by a teaching set of 20 low-dose CTs with examples of different types and sizes of opacities. Radiographers were then evaluated on further training subsets with feedback between subsets by local radiologists on up to 60 further CTs. Finally, radiographers and radiologists were required to achieve at least 80% sensitivity on a test set of 25 CTs. By the time of this study, each radiographer had also already read over 100 UKLS CTs prospectively as part of the previous investigation [20].

### Concurrent reading workflow

In concurrent reading, the following steps were performed:

1. Each CT was first read by a radiographer on a Leonardo workstation (Siemens Medical Solutions, Erlangen, Germany) using a commercially available software package capable of performing semi-automated volumetric nodule segmentation (Syngo LungCare, version Somaris/5 VB 10A). The radiographer then uploaded his or her report to the UKLS database under a personal login.
2. The radiographer’s stored nodule recordings (in the form of a DICOM structured report, or DICOM SR) were then made available to the reading radiologist. For each recording, the radiologist had one of three options. He could *accept* a particular finding if he agreed with it, and leave the radiographer recording unmodified. He could *reject* a finding if he disagreed with it (i.e. he thought the finding did not represent a nodule), in which case he would delete the recording. Finally, he could *amend* the recording if he agreed that the finding represented a nodule, but disagreed with its categorisation (for example, he thought it was a subsolid nodule where a radiographer had classified it as solid), in which case he would re-report the nodule using an electronic soft-copy entry proforma (Artex VOF, Logiton, The Netherlands) and copy and paste his own report into the nodule report. The radiographer was not present when the radiologist was performing this interpretation.
3. After reviewing the radiographer’s recordings, the radiologist performed another search to identify any additional nodules missed by the radiographer.
4. The radiologist then saved his recordings as a new DICOM SR, and uploaded this report to the UKLS database.

**Fig. 1:**
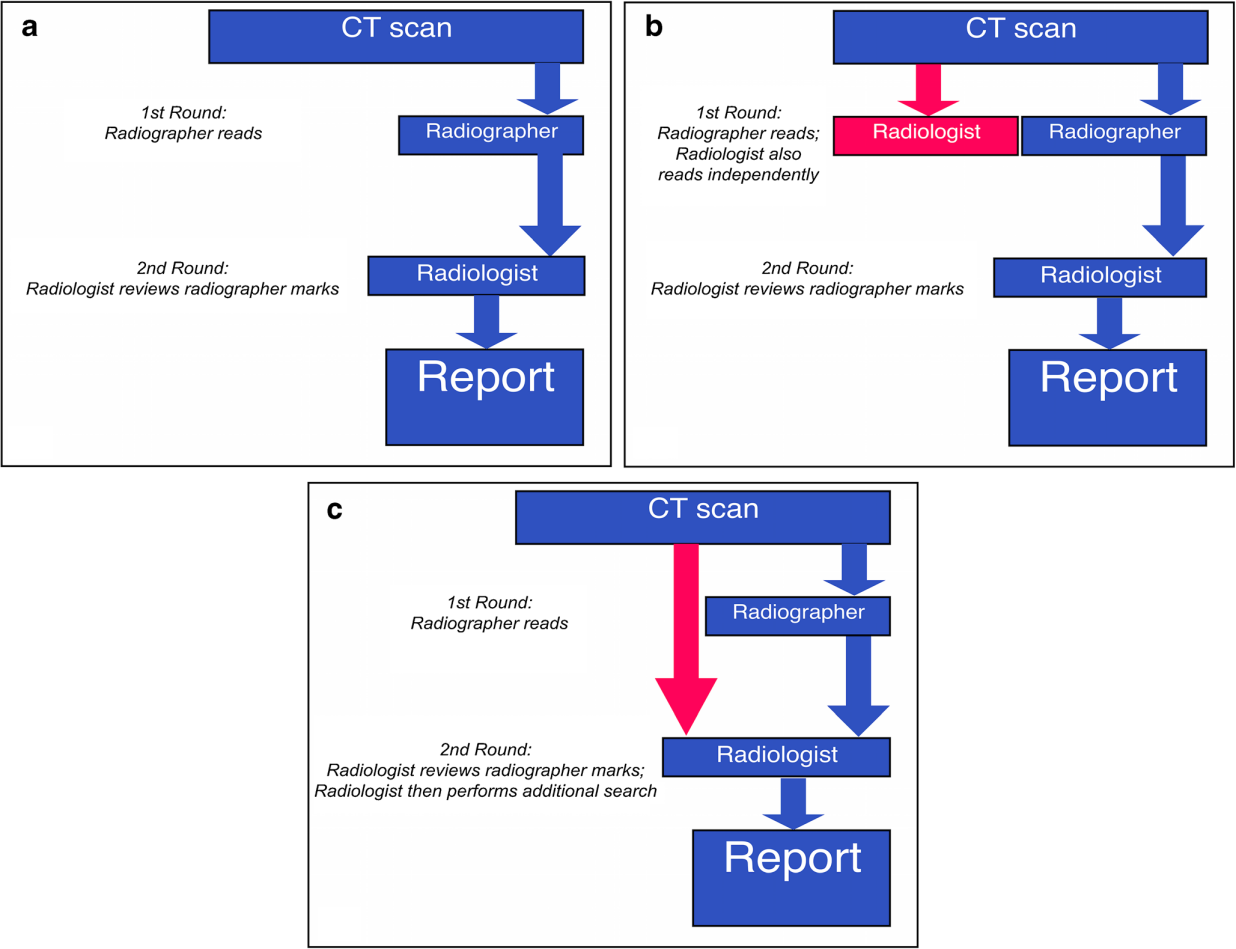
The process of reading in first (**A**), second (**B**) and concurrent (**C**) reading with radiographers

In this way, a given radiologist still only performed a single reading of the CT. Figure 1 summarises the concurrent reading process as compared to the first reading and the second reading.

### Selection of CT studies for independent single reading versus concurrent reading

It was decided that the most practical strategy for comparing the concurrent reading and independent single (i.e. unaided) reading performance for each radiologist would be for him to read a number of CTs using one method, and then to switch to the other method after 2 months. In this way, the radiologist would be reading in actual screening conditions, potential variability in CT studies (such as the number and type of nodules per CT) would be adjusted for, and central and local-site radiologists could all be assessed. As such, for the first 2 months (June-August), the local radiologists (Radiologists A and B) were instructed to read independently, while the central radiologists (Radiologists C and D) read concurrently with Radiographers 3 and 4. The reading methods were then switched between the sites over the second 2 months (August-October), with the central radiologists reading independently and the local radiologists reading concurrently with their respective radiographers (i.e. Radiologist A reading with Radiographer 1 and Radiologist B reading with Radiographer 2). In other words, regardless of whether concurrent reading or independent reading was used, each CT scan was always read by two radiologists (one at the Local Site and one at the Central Site).

### Reading times

Radiologists and radiographers were instructed to record the method and the times, to the nearest minute, on the CTs they read. The reading time included both the time taken to interpret the CT (and, in the case of concurrent reading, to decide on radiographer recordings as well as to perform an independent search) and then to input the data into the UKLS database. The decision to record to the nearest minute, rather than second, was made so that a meaningful change in reading time (i.e. measurable in minutes, not seconds) that would translate into a potential real saving in an actual screening setting could be detected. Recording to the nearest minute was also less disruptive to reading workflow. Reading time recordings were saved together with the radiologists’ reports on the UKLS database.

### Reference standard

After both local and central readings had been performed, the radiologists at the central site (Radiologist C or D) reviewed all identified nodule candidates for each subject on the database to identify any discrepancies. Arbitration on discrepancies was provided at the central site by a thoracic radiologist with more than 20 years of experience, and the final consensus answer was recorded on the database. All agreed nodules were considered positive in the reference standard. The reference standard was thus composed of any nodules which had been identified and agreed on by both radiologists, as well as any nodule that had been identified by at least one radiologist, and subsequently ratified by the expert arbiter, including those radiographer-identified nodules that had been agreed to or amended by a radiologist during concurrent reading.

### Statistical analysis

For descriptive purposes, both the diameters and volumes of reference standard nodules as recorded on the UKLS database are given as medians and interquartile range between the 25th and 75th percentiles (interquartile ranges (IQRs)), as neither measurement followed a normal distribution according to the D'Agostino and Pearson omnibus test for normality.

The numbers of concurrently and independently read CTs were calculated for each radiologist. Comparisons of the number of reference standard nodules between concurrent and independent reading datasets for each radiologist were performed using the two-tailed Mann-Whitney test (a non-parametric distribution of nodules was assumed).

Sensitivity, the absolute number of false-positive detections (FPs) and average FPs per case (expressed as mean and standard deviation) were separately determined for the cohorts of CTs read independently and concurrently per radiologist. The sensitivity of each radiologist was calculated by dividing the number of true-positive nodules detected by the total number of nodules in the reference standard for the cases read by that radiologist. Average FPs per case were calculated by dividing the total number of FPs by the total number of cases read by that radiologist. Differences in proportions were compared using the Chi-squared test or Fisher’s exact test in the case of smaller sample sizes [23, 24] as appropriate. In a post hoc analysis, we also calculated the number and percentage (of the total number) of radiographer-detected opacities that were considered FPs by radiologists in concurrent reading, for each of the six combinations of radiographer and radiologist (two combinations at the Local Sites, and four combinations at the Central Site).

Differences in reading times between concurrent and independent reading for each reader were compared using the independent samples t-test. A further post hoc analysis to determine the correlation between numbers of nodules and reading time was subsequently performed using Spearman’s rank correlation, and differences in correlation coefficients analysed for statistical significance using Fisher’s r to z transformation. All analyses were performed using Medcalc (version 12.5.0.0, MedCalc Software, Mariakerke, Belgium). A *p* value of less than 0.05 was assumed to be statistically significant.

## Results

### Reference standard

A total of 369 LDCT studies were read during the study period. 123 (33.3%) of the 369 CT studies did not contain any nodules. The reference standard thus consisted of 694 nodules in the remaining 246 (67.7%) CT studies. The majority of CTs had one (87/369, 23.6%), two (60/369, 16.3%), three (33/369, 8.9%) or four (25/369, 6.8%) nodules. Across the 369 CT studies, the median number of nodules per CT was one, with a range of 0–17 nodules. The majority of reference standard nodules were solid nodules (648/694 nodules, 93.4%), with 11 (1.6%) part-solid and 35 (5.0%) pure ground-glass nodules. Reference standard nodules had a median diameter of 4.8 mm (IQR 2.4 mm), and a median volume of 39.7 mm^3^ (IQR 47.2 mm^3^).

### Number of cases and number of nodules by reading method

The number of cases read by each radiologist varied from 83 to 119 using independent reading and from 69 to 122 using concurrent reading (Table 1). However, there was no significant difference between the numbers of reference standard nodules per case in the independent versus concurrent reading cohorts for any of the four radiologists (Supplementary Fig. S1).

**Table 1:** Numbers of cases read by each radiologist using each reading method

		Radiologist			
		A	B	C	D
Reading method	Independent	83	119	94	84
	Concurrent	88	79	122	69
	*p* value	0.83	0.18	0.41	0.74

*p* values are for differences between the number of reference standard nodules per case between independent and concurrent reading, derived from the Mann-Whitney test

*Note:* The total number of independent reads performed (n = 380) is higher than the total number of concurrent reads (n = 358) as there was a small number of scans that were read using independent reading by both local- and central-reading radiologists immediately around the time of the switchover after the first 2 months

### Sensitivity of radiologists

The overall sensitivity of each radiologist for the different reading methods is detailed in Table 2. The mean sensitivity for radiologists reading independently was 77.5 ± 11.2%, increasing to 90.8 ± 5.6% with the use of concurrent reading. For all but one radiologist (Radiologist D), statistically significant higher sensitivity was achieved with concurrent reading compared to independent reading.

**Table 2:** Sensitivity of radiologists for each reading method

		Radiologist			
		A	B	C	**D**
Reading method	Independent	78.9	79.8	62.2	89.2
	Concurrent	90.4	98.2	84.5	90.1
	Difference	11.5	18.4	22.3	0.9
	*p* value	**0.01**	**<0.0001**	**<0.0001**	0.97

Except for *p* values, figures shown are percentages

*p* values are those derived from the Chi-square test

*p* values in bold indicate statistically significant results

### False-positive detections (FPs)

**Fig. 2:**
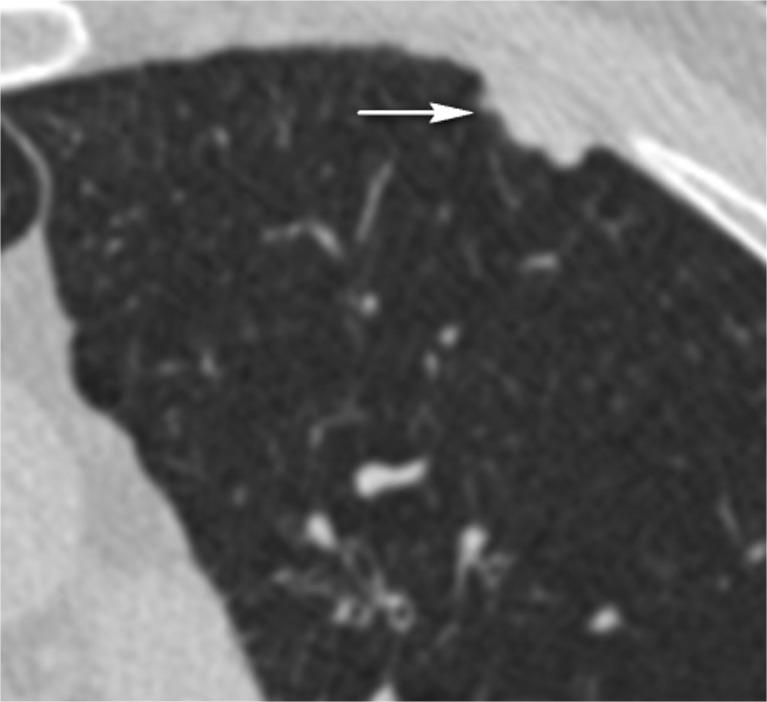
Example of a left upper lobe lesion (white arrow) identified on CT by a radiographer, but dismissed by the concurrent reading radiologist as a false-positive finding. The patient also had other calcified pleural plaques (not shown) consistent with previous asbestos exposure

There was a wide variation in the average FPs per case. While the overall mean of average FPs per case increased from 0.33 ± 0.20 with independent reading to 0.60 ± 0.53 with concurrent reading, average FPs per case ranged between 0.06 and 1.38, increasing with concurrent reading for Radiologists A, B and C (and statistically significant for Radiologists B and C), but decreasing for Radiologist D (Table 3). The percentage (of the total number) of radiographer findings considered false positive by the concurrently reading radiologist varied from 0.0 to 19.8% (Supplementary Table S1 and Fig. 2). For completeness, the number and percentages of reference standard nodules that were missed by radiographers, and that were detected but incorrectly categorised by radiographers, are illustrated in Supplementary Tables S2 and S3, respectively.

**Table 3:** Average false-positive detections (FPs) per case for each reading method

		Radiologist			
		A	B	C	D
Reading method	Independent	0.31 ± 0.75	0.47 ± 1.10	0.06 ± 0.25	0.48 ± 0.96
	Concurrent	0.37 ± 0.65	1.38 ± 1.46	0.21 ± 0.61	0.42 ± 0.76
	Difference	0.06	0.91	0.15	-0.06
	*p* value	0.56	**<0.001**	**0.03**	0.69

A negative difference indicates a lower average FP per case with concurrent compared to independent reading

*p* values are those derived from the independent samples t-test

*p* values in bold indicate statistically significant results

### Reading times

Radiologists A, B, C and D submitted their reading times for 100%, 77%, 75% and 90% of their concurrently read cases, respectively, and for 83%, 71%, 85% and 89% of their independently read cases, respectively. The mean reading times per case for concurrent reading ranged from 6.2 min to 8.6 min, compared to 7.0 to 12.4 min for independent reading (Table 4).

**Table 4:** Mean reading times of radiologists for each reading method

Reading method	Radiologist			
	A	B	C	D
Independent	12.4 (11.1,13.5)	8.8 (7.8, 9.8)	7.0 (6.5, 7.5)	8.3 (7.4, 9.3)
Concurrent	8.6 (7.9, 9.3)	6.2 (5.5, 7.0)	6.9 (6.4, 7.4)	7.0 (6.2, 7.8)
Difference	−3.8	−2.6	−0.1	−1.3
*p* value	**<0.0001**	**0.0001**	0.65	**0.03**

Numbers shown are time in minutes, except for *p* values

Numbers in parentheses are 95% confidence intervals for the mean

A negative difference indicates a shorter time with concurrent compared to independent reading

*p* values are those derived from the independent samples t-test

*p* values in bold indicate statistically significant results

Concurrent reading was faster than independent reading for all radiologists, but this increase in reading speed was not statistically significant for Radiologist C. Furthermore, the maximum decrease in mean reading time was just under 4 min (Radiologist A).

### Relationship between number of nodules per CT and reading time

The finding that concurrent reading was quicker despite there being no significant difference between the number of reference standard nodules per CT led to a post hoc analysis to investigate the relationship between the number of nodules per CT and reading time. As could be expected, there was a significant correlation between the number of reference nodules per subject and the time taken for each CT, and for Radiologists A, C and D this correlation was stronger for independently read CTs (Table 5). However, the strengthening of this correlation was only statistically significant for Radiologist D (0.766 for independent reading vs. 0.523 for concurrent reading, p = 0.01).

**Table 5:** Rank correlation between number of nodules per patient and time taken

Reading method	Radiologist			
	A	B	C	D
Independent	0.612 **(<0.0001**)	0.420 (**0.0001**)	0.432 (**0.0001**)	0.766 **(<0.0001**)
Concurrent	0.572 **(<0.0001**)	0.546 **(<0.0001**)	0.422 **(<0.0001**)	0.523 **(<0.0001**)
*p* value	0.71	0.34	0.94	**0.01**

Figures shown are Spearman’s coefficient of rank correlation (Spearman’s rho), and figures in parentheses are *p* values for significance level

*p* values in the final row are for significance levels of the difference between correlation coefficients for concurrent and independent reading per radiologist

Bold font indicates a statistically significant *p* value

## Discussion

The current investigation is, to our knowledge, the first evaluation of radiographers as concurrent readers for pulmonary nodule detection in CT lung cancer screening. It has shown that the sensitivity of the majority of radiologists in this study group improved by using radiographers as concurrent readers, with a statistically significant reduction in mean reading time, but accompanied by a simultaneous increase in FPs.

The improvement in sensitivity in this study was seen for all except the most sensitive radiologist, but the range of sensitivities of radiologists in this study was reassuringly comparable to that reported for radiologists [9, 16, 25–30]. As such, there is every reason to expect that concurrent reading with radiographers could improve the sensitivity of the majority of expert thoracic radiologists.

Of course, the increased average FPs per case that accompanied the increases in sensitivity with concurrent reading is once again a salutary reminder of the trade-off between sensitivity and ‘overcalling’ of nodules, especially for the two readers with the greatest sensitivity improvements (Radiologists B and C). Given the design of the present study, it is not possible to ascertain whether a radiologist may have designated the same false positives as a radiographer in the absence of a radiographer mark. Intuitively, however, it could be expected that the increase in FP detection is particularly due to radiographers identifying a large number of small opacities as nodules, and subsequent reticence of the radiologists to reject such marks once presented with them. Certainly, a previous study with CAD has shown that a radiologist’s confidence in designating a small nodule as positive was enhanced when presented with a CAD mark, even if it did not improve overall accuracy [31].

It is important to put the increase in average FPs per case into perspective when considering concurrent reading with radiographers as an alternate strategy to independent reading, as compared to CAD. CAD results in average FPs per case of 3.7–4.15 when including nodules as small as 3 mm, the size-cut off used for inclusion in the UKLS and the present study [18, 32]. This rate is much higher than the highest rate with concurrent reading in the present study (Radiologist B, 1.38). Nevertheless, the consequences that can be triggered by false positives (such as increased number of CTs requiring arbitration, follow-up and increased anxiety to patients) must be acknowledged.

Concurrent reading also proved statistically significantly faster than independent reading for three of the four radiologists. However, the statistical significance of the decrease in time may not translate into a clinically relevant time-saving benefit for every radiologist; in this study, Radiologist A would save just under 4 min per subject with concurrent reading, whereas Radiologist C was hardly affected by it. Nevertheless, when extrapolated to, for example, the 187,500 or so CTs per year that would need to be read in a UK national screening programme, a time saving of 4 min per CT for each radiologist becomes significant. More importantly, concurrent reading did not increase the mean reading time.

Three studies [17–19] have evaluated the use of CAD (rather than radiographers) as an additional reader for lung nodule detection. These studies assessed CAD as a second reader and as a concurrent reader, but with differing results. For instance, Beyer et al. [17] found that, despite concurrent reading with CAD being faster, pooled sensitivity was either lower or equivalent to independent reading, possibly because increased sensitivity with CAD-aided reading was offset by the radiologist tending to miss more nodules in the shorter reading time. The increased radiologists’ sensitivity with correspondingly more FPs in our study is at odds with Beyer et al., as it signals, if anything, an increased rather than decreased vigilance when using radiographers as concurrent readers. In contrast, Matsumoto et al. have recently demonstrated that concurrent reading with CAD was not only faster but could significantly improve sensitivity [19]. As has been highlighted in previous investigations [12, 13], such improved sensitivity is probably because the strengths of CAD and human readers are complementary; radiologists possibly have a superior sensitivity to CAD in hilar and subpleural areas, while CAD is more sensitive in central and perihilar regions. That said, the interactions between two human readers in the concurrent reading setting – that is, the radiologist and radiographer – are almost certainly more complex and require more detailed evaluation in future studies.

The present study has some limitations. The concurrent and independent reading cohorts consisted of different patients. However, this was the most practical way of performing this study prospectively, with cases being read under actual reporting rather than experimental conditions. Furthermore, it is reassuring that the number of reference standard nodules per subject between reading methods was not different for any of the four radiologists (i.e. the level of ‘difficulty’ was similar for both cohorts). The strategy of switching between reading methods would also have mitigated variations in each radiologist’s performance during one or other period.

Rather than have a separate individual recording time, each reader recorded their times. We recognised that this would result in reading times not being documented for some cases, but concluded that such a strategy best reflected ‘real-time’ reading practice. Put another way, it is possible that the presence of an additional overlooking observer responsible for measuring reading times, could affect reading performance. We also decided at the outset to record times to the nearest minute, and not second. A greater statistically significant reduction in time could potentially have been seen if reading times had been recorded to the second, but it was important in this study to detect time reductions that would translate into clinically meaningful reductions, i.e. minutes not seconds. There was also no way to blind each radiologist to the reading method he was using, by definition, and so there could have been a selection bias of the radiologists in choosing which cases they recorded times for. Again, this was the most practical way to record timing given the large numbers of CTs in our study. Finally, like all studies of human observer performance, the present results potentially reflect the performance and interaction of the radiologists and radiographers over a defined time period; with accumulated experience and changes in a particular radiologist-radiographer dynamic over time, such performance metrics could conceivably vary.

In conclusion, this study demonstrated that radiologists’ sensitivity in lung nodule detection could be improved with the use of radiographers as concurrent readers. An increase in FPs with radiographer-assisted concurrent reading occurred, but this increase was still below that reported for CAD systems. Concurrent reading with radiographers was also faster than single reading, but on a per-case basis the time saved was relatively modest.

## Electronic supplementary material

Below is the link to the electronic supplementary material.

ESM 1 (DOCX 23 kb)

Supplementary Fig. S1 (GIF 15 kb)

High resolution image (TIF 583 kb)

## Abbreviations

CAD: Computer-aided detection
CT: Computed tomography
DICOM: Digital Imaging and Communications in Medicine
DICOM SR: DICOM Structured Report
FPs: Absolute number of false-positive detections
UKLS: UK Lung Cancer Screening
