# Supplementary material for: The impact of trained radiographers as concurrent readers on performance and reading time of experienced radiologists in the UK Lung Cancer Screening (UKLS) trial
Source: Eur Radiol. 2017 Jun 22;28(1):226–34. doi: 10.1007/s00330-017-4903-z (PMC5717117; doi:10.1007/s00330-017-4903-z)
Supplement: Supplementary file 1 — (DOCX 23 kb) [file 330_2017_4903_MOESM1_ESM.docx]

The Impact of Trained Radiographers as Concurrent Readers on Performance and Reading Time of Experienced Radiologists in the UK Lung Screening (UKLS) trial: Supplementary Material

**Participation in the UKLS** **trial**

Briefly, subjects aged 50-75 years were approached with a questionnaire to determine lung cancer risk. Those with an estimated risk of at least 5% of developing lung cancer in the next 5 years (using the Liverpool Lung project risk model) (1) were invited to enrol in the trial. The pilot phase of the trial has randomised 2028 subjects to CT screening at two participating sites (Local Site A and Local site B), and 2027 subjects to no screening, between 2011 and 2013 (2).

**Ethical Approval**

Ethical approval for the UKLS trial was granted by the National Research Ethics Service Committee, NHS R&D, the National Information Governance Board for Health and Social Care, and the Administration of Radioactive Substances Advisory Committee (ARSAC) (application no. 0521, reference ECC 2-02(a)/2011, approved 15.03.2011). In addition, all participating sites had undergone site-specific assessment conducted by their local R&D department. Informed consent had been obtained from all participants. In consenting to the trial, participants also consented to further investigations, treatment, follow-up and data collection resulting from the trial, including permission to use data for future research.

**CT imaging protocol**

At both sites, examinations were performed on 16-row multi-detector CT systems (Sensation-16, Siemens Medical Solutions, Forchheim, Germany) in spiral mode, with thin collimation (0.5 – 0.625mm) and pitch of 0.9-1.1. Images were acquired at maximal inspiration during breath-holding, after appropriate instruction of the subjects, and without intravenous contrast. Exposure factors were tailored to patient height and weight, to achieve CT dose index (CTDIvol) between 0.8 and 3.2 mGy, with an effective radiation dose below 2 mSv. All appropriate dose modulation was used according to manufacturer’s guidelines and local practice. Data were reconstructed at 1.0 mm slice thickness with 0.7 mm reconstruction increment, using a moderate spatial frequency kernel.

**Classification of nodules**

The UKLS categorised nodules according to diameter and volume, as described previously (2). An electronic database entry proforma (Artex VOF, Logiton, Netherlands) was used for recording nodules.

**CT reading method**

All studies were read on a workstation (Leonardo; Siemens Medical Solutions, Erlangen, Germany) using a commercially available software package capable of performing semi-automated volumetric nodule segmentation (Syngo LungCare, version Somaris/5 VB 10A). Studies loaded into LungCare were presented in the following manner: 2 x 2 viewing partition with a default window setting level -500 HU, width 1500 HU, default display of transverse maximum intensity projections (MIPs) at 10mm thickness in cine mode, 1mm-collimation transverse images, 0.7mm-collimation coronal images, and a panel for display of semiautomatic volumetric segmentation analysis if performed. Readers could alter MIP thickness and window settings.

Semi-automated volumetry was used in all cases where segmentation of a nodule could be reliably performed, and is similar to the technique described in other studies using this software (3). First, the reader marks a candidate nodule with a mouse click. Then the programme automatically defines a volume of interest around the candidate nodule, which can be further analysed by using volume-rendered displays or a coronal reformation. The candidate nodule can then be either approved or discarded. The evaluation of a nodule with a second mouse click initiates an automated volume measurement programme. In cases of unreliable or impossible segmentation, manual measurements were performed.

Once a reading for a particular nodule had been completed, the information from the proforma was copied and pasted into a structured DICOM report in an Extensible Markup Language (XML) format. The XML file was then uploaded to the patient’s record on the UKLS database (a secure web-based database) via a PC with web access. The XML file contained information regarding a nodule’s size, table position location, lung and segment location, category and volume.

**Supplementary Figures and Tables**

Supplementary Figure Legend:

Supplementary Figure S1. No. of reference standard nodules per case for each reading method for Radiologists A-D.

CR= concurrent reading, IR= independent reading.

**Supplementary Table S1. Radiographer findings considered false positive (FP) by radiologists in concurrent reading.**

| **Combination of reading radiographer and radiologist in concurrent reading** | **Total number of opacities detected by radiographer** | **Number (%) of radiographer opacities designated FP by radiologist reading concurrently ^a^** | **Number (%) of radiographer opacities designated FP by radiologist that were subsequently also considered FP in the reference standard ^b^** |
| --- | --- | --- | --- |
| **1-A** | 172 | 21 (12.2) | 19 (90.4) |
| **2-B** | 247 | 9 (3.6) | 8 (88.9) |
| **3-C** | 147 | 18 (12.2) | 11 (61.1) |
| **3-D** | 74 | 3 (4.1) | 2 (66.7) |
| **4-C** | 81 | 16 (19.8) | 9 (56.3) |
| **4-D** | 15 | 0 (0.0) | Not applicable |

^a^ Numbers given in parentheses are the percentage of all radiographer-detected opacities that were designated false positive by a radiologist.

^b^ Numbers given in parentheses are the percentage of all radiographer-detected opacities that were designated FP by a radiologist, and also subsequently considered false positive in the reference standard.

**Supplementary Table S2. No and percentage of reference standard nodules missed by radiographers.**

| **Radiographer** | **No. of reference standard nodules in cases read by radiographer** | **No. (%) of references standard nodules missed by radiographer** |
| --- | --- | --- |
| **1** | 241 | 46 (19.1) |
| **2** | 312 | 65 (20.8) |
| **3** | 393 | 74 (18.8) |
| **4** | 280 | 89 (31.8) |

**Supplementary Table S3. No. and percentage of reference standard nodules that were detected but incorrectly categorised by radiographers.**

| **Radiographer** | **No. of reference standard nodules detected radiographer** | **No. (%) of references standard nodules detected by radiographer that were incorrectly categorised** |
| --- | --- | --- |
| **1** | 195 | 40 (20.5) |
| **2** | 247 | 69 (27.9) |
| **3** | 319 | 73 (22.9) |
| **4** | 191 | 29 (15.2) |

**References**

1. Cassidy A, Myles JP, van TM, et al. (2008) The LLP risk model: an individual risk prediction model for lung cancer. Br J Cancer 98:270-276

2. Field JK, Duffy SW, Baldwin DR, et al. (2016) UK Lung Cancer RCT Pilot Screening Trial: baseline findings from the screening arm provide evidence for the potential implementation of lung cancer screening. Thorax 71:161-170

3. Gietema HA, Wang Y, Xu D, et al. (2006) Pulmonary nodules detected at lung cancer screening: interobserver variability of semiautomated volume measurements. Radiology 241:251-257
