# Supplementary figures and images for: The impact of trained radiographers as concurrent readers on performance and reading time of experienced radiologists in the UK Lung Cancer Screening (UKLS) trial
Source: Eur Radiol. 2017 Jun 22;28(1):226–34. doi: 10.1007/s00330-017-4903-z (PMC5717117; doi:10.1007/s00330-017-4903-z)

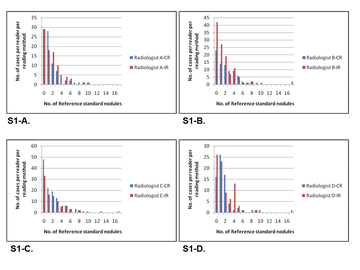

Supplement: Supplementary file 2 — (GIF 15 kb) [file 330_2017_4903_Fig3_ESM.gif]

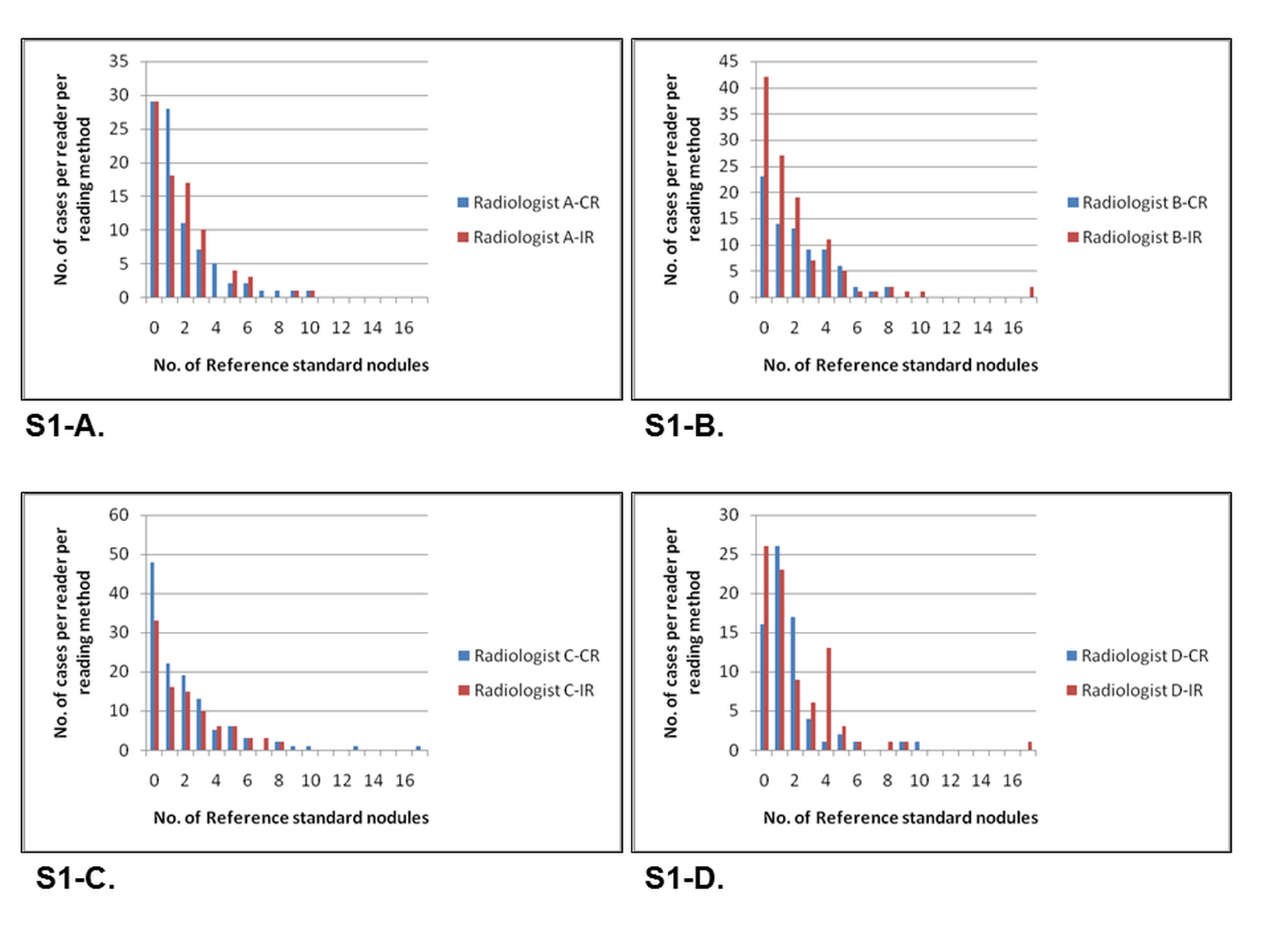

Supplement: Supplementary file 3 — High resolution image (TIF 583 kb) [file 330_2017_4903_MOESM2_ESM.tif]
